# Draft Genome Sequences of Azospirillum brasilense Strains Ab-V5 and Ab-V6, Commercially Used in Inoculants for Grasses and Legumes in Brazil

**DOI:** 10.1128/genomeA.00393-18

**Published:** 2018-05-17

**Authors:** Mariangela Hungria, Renan Augusto Ribeiro, Marco Antonio Nogueira

**Affiliations:** a Embrapa Soja, Soil Biotechnology Laboratory, Londrina, Paraná, Brazil; b CNPq, SHIS QI 1 Conjunto B, Brasília, Federal District, Brazil

## Abstract

Azospirillum brasilense strains Ab-V5 and Ab-V6 are largely used in commercial inoculants for grasses and legumes in Brazil. Their genomes were estimated at 6,934,595 and 7,197,196 bp, respectively, and encompass genes related to nitrogen fixation, synthesis of phytohormones, and environmental adaptation. Although the strains differ in phenotypic properties, their genomes are highly similar.

## GENOME ANNOUNCEMENT

The use of inoculants carrying plant growth-promoting bacteria (PGPB) is increasing, stimulated by a global search for a more productive but sustainable agriculture, and the genus Azospirillum comprises one of the most studied and used PGPB. Brazil has a long tradition of studying Azospirillum spp. (1), but it was only in 2009 that the first strains started to be used in commercial inoculants (2). Since then, two strains―Ab-V5 (CNPSo 2083) and Ab-V6 (CNPSo 2084)―identified by our research group (2) have been used in inoculants for grasses (maize [Zea mays L.], wheat [Triticum aestivum L.] [2, 3], and pastures with brachiaria [Urochloa spp.] [4]), and for coinoculation of legumes (soybean [Glycine max L. Merr.]) and common bean (Phaseolus vulgaris L.) (5, 6). In the 2017 to 2018 crop season, about 5 million doses of inoculants carrying strains Ab-V5 and Ab-V6 were commercialized in Brazil, and their use is expanding in neighboring countries. Here, we report the draft genomes of these two strains.

DNA extraction and paired-end sequencing on the MiSeq platform (Illumina) were performed as described before (7), and sequences were assembled with the A5-miseq pipeline (*de novo*). Shotgun sequences allowed genome coverages of 245- and 60-fold for Ab-V5 and Ab-V6, respectively. The genome of strain Ab-V5 was estimated at 6,934,595 bp, with a G+C content of 68.4 mol%, and the genome of Ab-V6 was estimated at 7,197,196 bp, with a G+C content of 68.3 mol%; both genomes were assembled with 63 contigs. The sizes are within the range of other sequenced A. brasilense strains, including CBG497 (6,473,208 bp) (8), Az39 (7,391,279 bp) (9), and Sp245 (7,530,241 bp) (8). A. brasilense strains usually carry plasmids (8, 9), and from Eckhart gel electrophoresis, we observed that Ab-V5 and Ab-V6 have at least two common plasmids (of about 285,000 and 100,000 bp) and that Ab-V6 carries an extra plasmid of about 150,000 bp. Besides that, the Ab-V5 and Ab-V6 genomes are highly similar, with an average nucleotide identity estimated at 100%.

Sequences were submitted to the Rapid Annotations using Subsystems Technology (RAST) server (10), and 6,349 DNA coding sequences (CDSs) were identified in Ab-V5 and 6,625 CDSs in Ab-V6, classified into 510 and 519 subsystems, respectively; 53 and 54% of the CDSs were not classified in any subsystem, respectively. Both strains carry similar *nif* and *fix* genes that confer their ability to fix atmospheric nitrogen. Although the strains differ in their capacity to synthesize phytohormones (11, 12), both share the same genes related to the synthesis of auxins. One important feature of Ab-V5 and Ab-V6 is their capacity to induce genes related to tolerance of biotic and abiotic stresses in plants (11, 13), and the strains also carry several stress response genes, the majority of which are related to oxidative stresses. The reported niche adaptation of A. brasilense (8) might be attributed to a whole range of genes related to motility and chemotaxis, phosphorus metabolism, and resistance to antibiotics, among others. As reported before (12), we found several copies of genes encoding quorum-sensing LuxR but not LuxI proteins; in addition, there were no genes related to type III secretion systems.

### Accession number(s).

The whole-genome shotgun project for strain Ab-V5 has been deposited at DDBJ/EMBL/GenBank under the accession number POQV00000000 (accession number SUBID SUB3474034, BioProject PRJNA429443, and BioSample SAMN08346097); the version described in this paper is the first version, POQV01000000. The whole-genome shotgun project for strain Ab-V6 has been deposited at DDBJ/EMBL/GenBank under the accession number POTD00000000 (accession number SUBID SUB3488520, BioProject PRJNA429631, and BioSample SAMN08354664); the version described in this paper is the first version, POTD01000000.
